# Nucleus-forming vibriophage cocktail reduces shrimp mortality in the presence of pathogenic bacteria

**DOI:** 10.1038/s41598-023-44840-x

**Published:** 2023-10-19

**Authors:** Khrongkhwan Thammatinna, Ammara Sinprasertporn, Ampapan Naknaen, Thanadon Samernate, Jiratchaya Nuanpirom, Parinda Chanwong, Kunlaya Somboonwiwat, Joe Pogliano, Ponsit Sathapondecha, Jumroensri Thawonsuwan, Poochit Nonejuie, Vorrapon Chaikeeratisak

**Affiliations:** 1 Department of Biochemistry, Faculty of Science, Chulalongkorn University, Bangkok, Thailand; 2 Center of Excellence for Molecular Biology and Genomics of Shrimp, Department of Biochemistry, Faculty of Science, Chulalongkorn University, Bangkok, 10330 Thailand; 3 Songkhla Aquatic Animal Health Research and Development Center (SAAHRDC), Department of Fisheries, Songkhla, Thailand; 4 Institute of Molecular Biosciences, Mahidol University, Salaya, Nakhon Pathom Thailand; 5 Center for Genomics and Bioinformatics Research, Division of Biological Science, Faculty of Science, Prince of Songkla University, Hat Yai, Songkhla Thailand; 6 Division of Biological Sciences, University of California San Diego, La Jolla, CA USA

**Keywords:** Microbiology, Bacteriophages, Phage biology

## Abstract

The global aquaculture industry has suffered significant losses due to the outbreak of Acute Hepatopancreatic Necrosis Disease (AHPND) caused by *Vibrio parahaemolyticus*. Since the use of antibiotics as control agents has not been shown to be effective, an alternative anti-infective regimen, such as phage therapy, has been proposed. Here, we employed high-throughput screening for potential phages from 98 seawater samples and obtained 14 phages exhibiting diverse host specificity patterns against pathogenic VP_AHPND_ strains. Among others, two *Chimallinviridae* phages, designated Eric and Ariel, exhibited the widest host spectrum against vibrios. In vitro and in vivo studies revealed that a cocktail derived from these two nucleus-forming vibriophages prolonged the bacterial regrowth of various pathogenic VP_AHPND_ strains and reduced shrimp mortality from VP_AHPND_ infection. This research highlights the use of high-throughput phage screening that leads to the formulation of a nucleus-forming phage cocktail applicable for bacterial infection treatment in aquaculture.

## Introduction

Global food security has deteriorated rapidly in recent years due to a number of factors, of which climate change stands out as a major factor. Specifically, adverse effects of climate abnormalities were observed across all agricultural sector value chains^1,2^. The yield of many agricultural goods is severely impacted by the increased possibility of major disease outbreaks brought on by unpredictable weather patterns, and aquaculture products are not spared from this inevitable fate. In many coastal food exporting countries, shrimp was one of the most crucial fishery products^3,4^, contributing to economic prosperity and uplifting millions of people's lives. In recent years, however, the shrimp industry has been confronted with an increasing number of disease epidemics, especially acute hepatopancreatic necrosis disease (AHPND)^5–7^, resulting in a precipitous decline in shrimp production.

AHPND is typically characterized by the unusual and acute mortality of shrimp within the first 35 days in cultivation ponds and the mortality rate could easily reach 100% in larvae stage^8^. The disease is caused by the infection of a gram-negative rod-shaped bacteria *Vibrio parahaemolyticus*^9^. The presence of *V. parahaemolyticus* is dependent on many environmental conditions, such as pH, temperature, and salinity^9,10^. It is detected year-round in areas, in which the water temperature is above 15 °C, and increases in quantity are detected with increased temperatures^11^. Rising ocean temperatures due to global warming significantly propel the increased range and frequency of infection with *V. parahaemolyticus*^12^. In 2009, a new strain called *V. parahaemolyticus* AHPND (VP_AHPND_) emerged in southern China and spread into major shrimp production hubs in east and southeast Asian countries^13^, causing a long-lasting economic impact to the region. To prevent large-scale losses, an abundance of antibiotics is used due to their low cost and accessibility^14^. However, the misuse and overuse of antibiotics are promoting the spread of antibiotic resistance in bacteria^15^. The extensive misuse of antibiotics in aquaculture has also caused prolific increases in resistant strains and multi-antibiotic resistance in shrimp pathogens^16^. In addition, the resistance could be transferred to other bacterial species, including human pathogens, leading to uncontrollable resistome overspill and eventually severe consequences for human health^17^. This issue urges the need for effective alternative therapeutics that could mitigate the adverse effect on antibiotic resistance.

One of the most promising antibiotic alternatives is bacteriophage therapy, the method in which evolutionary races between two organisms are exploited for therapeutic purposes^18,19^. Phages, viruses infecting their specific bacterial host, typically enter either a lytic or lysogenic (temperate) life cycle^20^. Since one of the key events during the lysogenic cycle is characterized by the insertion of a phage genome and maintaining its genetic material within the host without destroying the host cell DNA^20,21^, lytic phages are highly preferred for use as therapeutic agents. In addition, integration of the phage genome could potentially contribute to more virulent bacterial strains if genes encoding virulence factors are carried within the genome^22^. The use of lytic phages has many advantages over the use of antibiotics, including auto-dosing, biofilm penetration, and high specificity, which disallows cross-infection, thereby minimizing its impact on bacterial community interference^20,23^. With these advantages, the use of phages and mixtures of phages, known as phage cocktails, for biocontrol agents in aquaculture recently came under the spotlight. In particular, phages targeting various pathogenic vibrios, including *V. harveyi*, *V. coralliilyticus*, *V. campbellii*, *V. anguillarum*, *V. alginolyticus*, *V. splendidus*, *V. cyclitrophicus*, and *V. parahaemolyticus*, have been extensively isolated and studied^24^, some of which demonstrated successful outcomes. Notably, a previous study showed that the use of phage cocktails in shrimp infected with pathogenic *Vibrio* sp. resulted in a 91.4% survival rate, a comparable outcome to that of antibiotic treatments at 91.6%^25^.

Here, we first developed a high-throughput phage screening method in order to swiftly screen for the vibriophages capable of infecting a collection of pathogenic VP_AHPND_ strains that were collected from local farms where the bacterial spread has been reported. The method was then used to screen through 98 seawater samples against the bacteria panel and led to the discovery of two virulent nucleus-forming vibriophages that were specific to a wide range of VP_AHPND_ strains. A cocktail derived from these vibriophages displayed high efficiency in both in vitro and in vivo experiments by suppressing the regrowth of bacteria and rescuing shrimp from infection within 24 h. Moreover, the vibriophages were able to colonize the digestive tract of shrimp, offering alternative aspects for prophylaxis against the bacteria in the future.

## Results

### Phage isolation by the high-throughput screening (HiTS) method leads to the discovery of two phages that exhibit a broad host range

**Figure 1:**
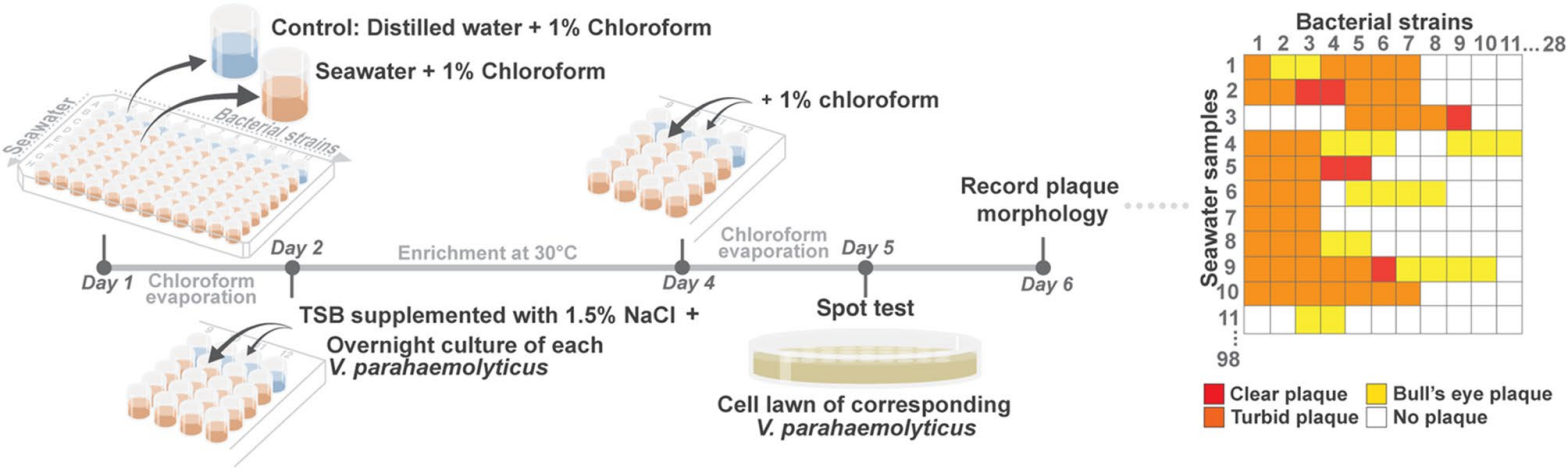
High throughput phage screening (HiTS) method for phages*,* with slight modifications, showing an integrated workflow for searching phages from 98 seawater samples and 28 bacterial hosts. Screening in a checkerboard manner can combine all samples with all bacteria of interest. The enrichments were examined whether phages are present by the spot test against corresponding bacterial hosts. Plaque morphologies are then recorded and represented by following colors; red for clear plaque, orange for turbid plaque, yellow for bull’s eye plaque, and white for no plaque, for further purification step. (See also Table S1).

Here, we employed the high-throughput screening (HiTS) method for phages^26^*,* with slight modifications (Fig. 1). A collection of *V. parahaemolyticus* strains (28 strains) from Songkla Aquatic Animal Health Research and Development Center (SAAHRDC) that were previously isolated from infected shrimp in local farms were used as parental hosts for extensive screening for phages in 98 different seawater samples. The enrichment cultures obtained from the method were then tested against corresponding *Vibrio* isolates to determine the presence of phages. The appearance of plaques on the bacterial cell lawns was recorded and counted as a hit. Out of 98 seawater samples, 72 samples revealed potential hits where putative plaques were observed (Table S1). The appearances of plaques from the identified hits included clear, turbid, and bull’s eye morphology. Since the clear plaque morphology was assumed to contain lytic phages, only 12 enrichment cultures that produced the clear plaque morphology were further processed for the purification step (Table S1; red). After the phage purification using their parental bacterial hosts, we eventually obtained 14 phages from the screen as designated: phiKT1015, phiKT1016, phiKT1017, phiKT1018, phiKT1019, phiKT1020, phiKT1021, phiKT1022, phiKT1023, phiKT1024, phiKT1025, phiKT1026, phiKT1027, and phiKT1028 (Table 1).

**Table 1:**
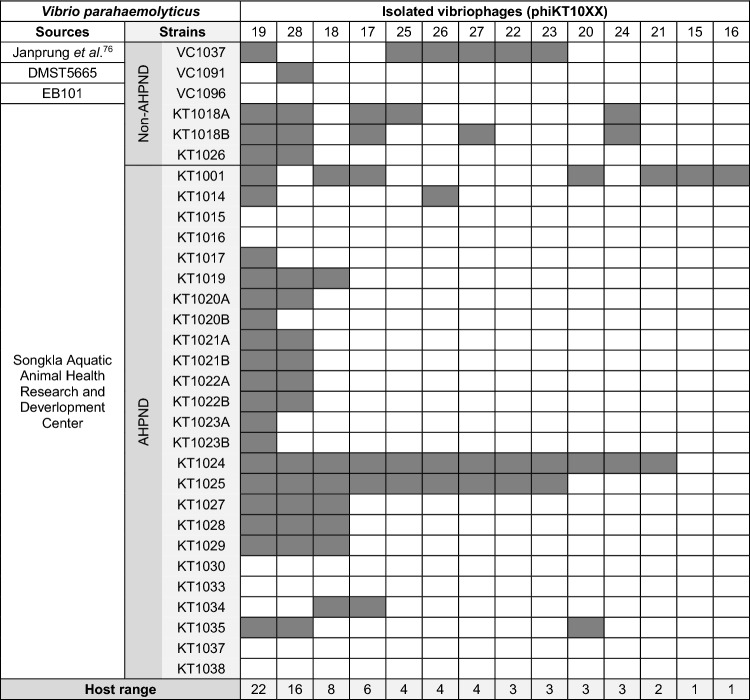
Host range determination of 14 isolated phages.

To determine the host spectrum of the isolated phages, various strains of *V. parahaemolyticus*, including non-AHPND and pathogenic AHPND strains, were used as the hosts. Grey and white boxes indicate appearance of plaque and no plaque, respectively. The total number of susceptible hosts to each phage was  recorded as shown in the last row of the table.

Since various VP_AHPND_ strains and their multidrug-resistant variants have spread widely in aquaculture, it is important to have effective phages that can kill a wide range of vibrios. To determine the host spectrum of these isolated 14 phages, we examined their killing activity against *V. parahaemolyticus* strains (Table 1). The isolated phages revealed differences in their specificity towards the bacteria, resulting in distinct host range patterns, which suggested that different phages are obtained from the screening method. Most of the isolated phages displayed a narrow spectrum towards the hosts (3–13%). Among them, a moderate host range was observed in phages phiKT1017 and phiKT1018 (19–26%) and the broadest host range was observed in phages phiKT1019 and phage phiKT1028 (52–71%) (Table 1). With the aim of formulating a phage cocktail that is applicable to a wide range of VP_AHPND_ strains, the two phages displaying a broad host range, phiKT1019 and phiKT1028, were then selected as candidates for the cocktail formulation to control VP_AHPND_.

### The phage candidates are effective and safe for use in biocontrol

**Figure 2:**
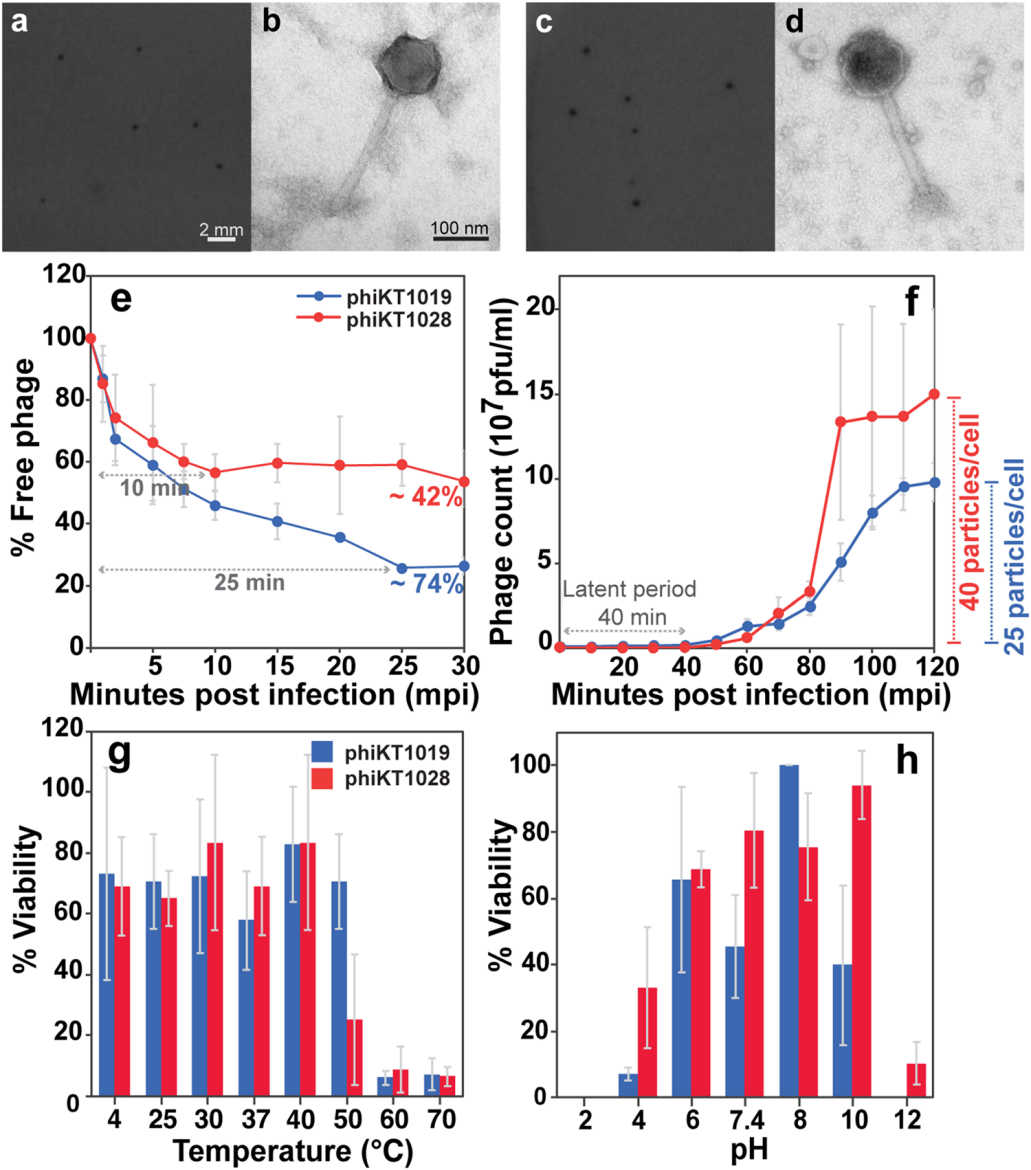
Characterization of morphological and biological properties of the selected phage candidates; phiKT1019 and phiKT1028. Plaque morphology of phage phiKT1019 (**a**) and phage phiKT1028 (**c**). Scale bars, 2 mm. Morphology of phages: phiKT1019 (**b**) and phiKT1028 (**d**) observed by TEM and negative staining. Scale bars, 100 nm. Biological studies of phages phiKT1019 (blue) and phiKT1028 (red); phage adsorption assay (**e**), one-step growth curve (**f**), and stability of phages at various temperatures (**g**) and pHs (**h**). The experiments were performed in at least 3 independent biological replicates and the data are represented as mean ± standard deviation.

To characterize whether the candidates are appropriate for use in biocontrol, phage biology and conventional phage kinetics of phages phiKT1019 and phiKT1028 were first conducted. Both phages were capable of lysing various *V. parahaemolyticus* strains (Table 1) and produced 0.5 mm-clear circular plaques on *V. parahaemolyticus* KT1018B cell lawns (Fig. 2a and c). The negative straining of phage structures under the Transmission Electron Microscope (TEM) revealed that these phages belonged to the order *Caudovirales* and the family *Myoviridae*. Both phages had a large hexagonal capsid (a diameter of 104.57 ± 7.31 nm in phiKT1019 and 129.25 ± 2.7 nm in phiKT1028) and a contractile tail (a length of 226.63 ± 12.50 nm in phiKT1019 and 232.35 ± 2.30 nm in phiKT1028) (Fig. 2b and d; n = 3). According to the adsorption assay, both phages had somewhat different adsorption rates on the bacterial host cells. PhiKT1019 exhibited a higher adsorption rate than phiKT1028, reaching approximately 74% of absorbed phages within 25 min. About 42% of phiKT1028, on the other hand, attached to the host within 10 min and remained at a similar level until 30 min (Fig. 2e). However, both phages required a similar latent period of approximately 40 min for maturation to reproduce around 25–35 particles per cell in phiKT1019 and phiKT1028, respectively (Fig. 2f). Under environmental factors, both phages appeared to be stable over a relatively wide range of temperatures and pHs. The phages remained active at temperatures ranging from 4 to 50 °C, but their infectivity drastically decreased when the temperatures were over 50 °C (Fig. 2g). Phage phiKT1028, however, was more tolerant of the extreme pHs than phiKT1019. PhiKT1028 remained infective (40–100%) at pHs ranging from 4 to 10, while phiKT1019 almost completely lost its activity (lower than 5%) at pH 4 (Fig. 2h).

**Figure 3:**
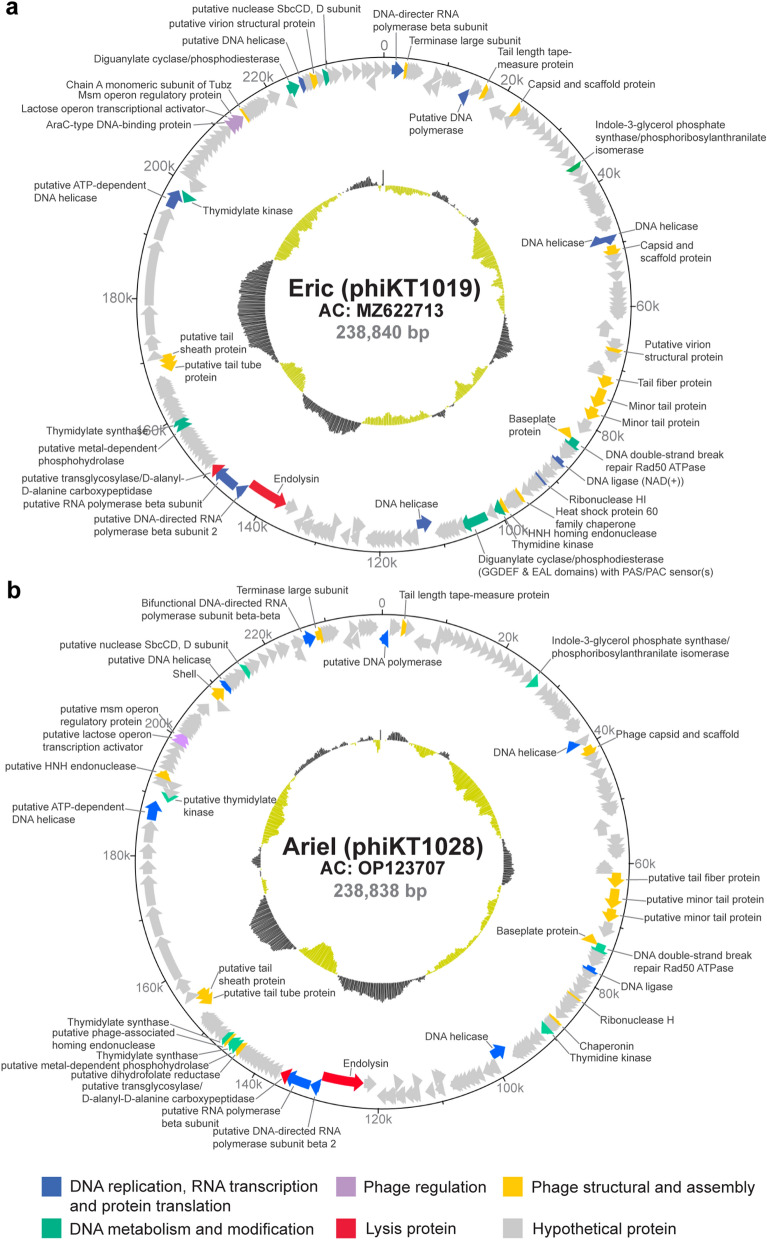
Genome maps of phages phiKT1019, designated as Eric (**a**), and phiKT1028, designated as Ariel (**b**). The accession numbers of each phage are MZ622713 and OP123707, and the data are available in NCBI database. The draft genome size of phages phiKT1019 and phiKT1028 are 238,840 and 238,838 base pairs long and the position (bps) are indicated on the outermost circle. The functional assignments of each open reading frames (ORFs) are represented by the following colors; blue for DNA replication, RNA transcription and protein translation, green for DNA metabolism and modification, purple for phage regulation, red for lysis protein, yellow for phage structure and assembly, and grey for hypothetical protein. Each arrow represents the ORF and its direction represents the gene arrangement in genomes (forward and reverse directions). The GC content of the genomes are indicated by the internal grey and green histograms.

To gain more insights into the phage candidates at the genetic level, we sequenced and analyzed the whole genomes of the phages phiKT1019 and phiKT1028. The whole genome sequencing of both phages resulted in more than 7 million reads of FastQ sequence after host genome subtraction. After genome assembly of the genomes with coverage over 700X, we obtained the whole genomes of the phages phiKT1019 and phiKT1028 (Fig. 3). Both phages had large double-stranded DNA genomes: with draft genome size of 238,840 bp for phiKT1019 and 238,838 bp for phiKT1028, encoding 218 open reading frames (ORFs) with 48.81% CG content in phage phiKT1019 and 209 ORFs with 44.83% CG content in phage phiKT1028 (Fig. 3a and b; Accession numbers MZ622713 and OP123707). Due to the size of the genome larger than 200 kbp, the dsDNA phages phiKT1019 and phiKT1028 are classified as giant or jumbophages. The annotated ORFs were assigned to the basic phage-functional modules as follows: (I) DNA replication, transcription, and protein translation; (II) DNA metabolism and modification; (III) Phage regulation; (IV) Phage structural and assembly; (V) Lysis-related proteins; and (VI) Hypothetical proteins (Fig. 3). We did not find any tRNA genes in these phages. Moreover, the genomes of both phages did not harbor any unwanted genes. Neither the genes related to putative toxins and antimicrobial resistance nor the lysogeny-related genes were found in these phage candidates. Since genome analysis suggested that both phages are lytic phages due to the absence of lysogeny-related genes, we further validated our genome annotation by lysogeny test (Figure S1). Our result revealed that, after mitomycin C activation of prophages in the phage-resistant isolates, none of the phage plaques were detected. This data suggested that these phages could not lysogenize the host and thus are obligated to enter the lytic cycle. Altogether, due to their capability of targeting a broad host spectrum, their resilience in harsh environments, and their virulent nature, phages phiKT1019 and phiKT1028 are potentially effective phages and safe for use in biocontrol.

### The phages phiKT1019 (Eric) and phiKT1028 (Ariel) are nucleus-forming vibriophages

**Figure 4:**
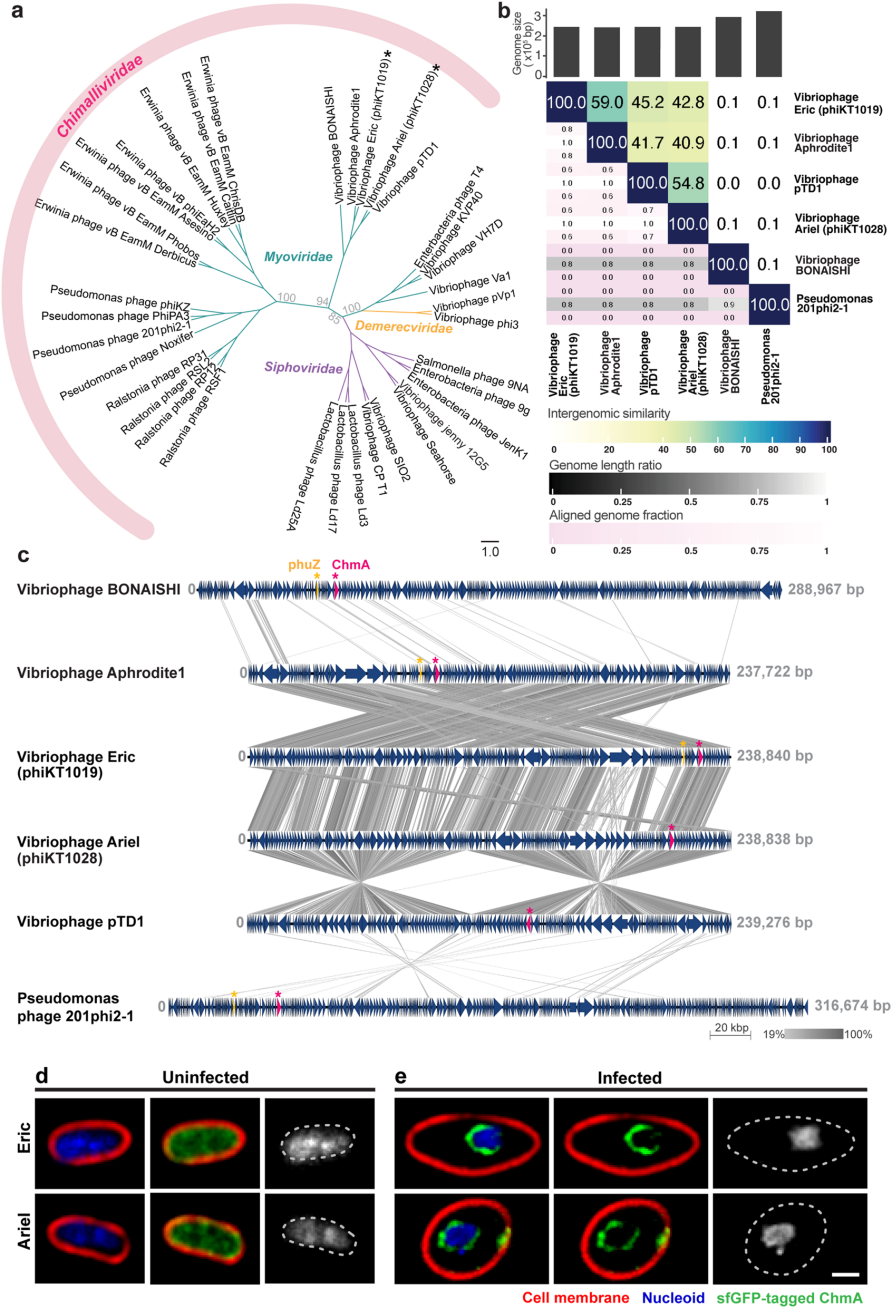
The phages phiKT1019 (Eric) and phiKT1028 (Ariel) are classified into the *Chimalliviridae* family and are nucleus-forming vibriophages. (**a**) Phylogenetic analysis of the relationship between genome of various selected phages from the database. Bootstrap values (%) of the tree as constructed by VICTOR and MEGA are shown at each main clade. The phage families, including *Chimalliviridae*, *Siphoviridae*, *Demerecviridae*, and *Myoviridae*, are included in the tree. Asterisks represent the branch of vibriophages Eric and Ariel. Scale bar represents the branch length of taxa. (**b**) VIRIDIC heatmap showing the intergenomic similarities of selected phages’ genomes; vibriophages BONAISHI, Aphrodite1, pTD1, Eric, Ariel, and *Pseudomonas* phage 201 Phi2-1. The intergenomic similarities between each pair of phage genomes are shown as percentage on the right half of the figure. The closely-relationship of the phages is represented by the dark blue color (scaled by color annotation of intergenomic similarity). (**c**) Genome comparison of 6 selected phages. The dark blue arrows represent the annotated ORFs with their directions. The ORFs encoding for PhuZ and ChmA are color-coded in yellow and pink, respectively. The similarity of phage genome regions is indicated by gradient shades of grey. (**d**) Single-cell level assay revealing the assembly of the phage nucleus during infection with vibriophages Eric and Ariel. Fluorescence images of uninfected *V. parahaemolyticus* KT1018B expressing sfGFP-tagged ChmA (upper panel, ORF203, lower panel, ORF180) and *V. parahaemolyticus* KT1018B expressing sfGFP-tagged ChmA infected with corresponding vibriophages at MOI_input_ 1 (upper panel, ORF203 infected with Eric, lower panel, ORF180 infected with Ariel). DNA was stained with DAPI (blue or grey) and cell membrane was stained with FM4-64 (red). Dashed lines indicate cell borders. Scale bars, 1 micron. (See also Figure S2).

To investigate the genetic relationship of the phages phiKT1019 and phiKT1028 with other phages that are available in the NCBI database, we first conducted a nucleotide blast search using our phage genomes as queries against the database. The Blastn search revealed that the genome of phage phiKT1019 was highly similar to that of vibriophage Aphrodite1 with 76.20% identity and 78% coverage, while the genome of phage phiKT1028 was highly similar to that of vibriophage pTD1 with 75.73% identity and 71% coverage. We then selected the closely and distantly related phages to both phages phiKT1019 and phiKT1028 as available in the database and constructed a phylogenetic tree to investigate the relationship among the selected phages. The tree showed that both phages phiKT1019 and phiKT1028 were grouped together into the clade of *Myoviridae*, further supporting the classification of viruses, based on the phage structure as observed previously by TEM (Figs. 2b, d, and 4a, asterisks). Interestingly, both phages were also genetically clustered together with the recently proposed *Chimalliviridae* family^27^, with a close genetic relationship with vibriophages BONAISHI, Aphrodite1, and pTD1 (Fig. 4a). The recently proposed *Chimalliviridae* family is a novel viral family whose phages share conserved core genes necessary for nucleus-based replication. To examine the similarity of our phages with other closely-related phages in the *Chimalliviridae* family, we used the VIRIDIC tool to evaluate the intergenomic similarity among the phages^28^. We found that phiKT1019 is most similar to vibriophage Aphrodite1 and phiKT1028 is most similar to vibriophage pTD1, with a similarity score of 59% and 54.8%, respectively. Based on the cutoff for identifying novel species (95%) and novel genus (75%)^28^, the intergenomic similarity values among the phages indicated that phages phiKT1019 and phiKT1028 are novel vibriophages in the *Chimalliviridae* family (Fig. 4b).

The fact that phiKT1019 and phiKT1028 belong to the *Chimalliviridae* family, which is possibly capable of forming a phage nucleus, is particularly interesting. It has been previously demonstrated that, during the replication inside the bacterial host, the nucleus-forming phages encode the chimallin (ChmA) protein with other accessory proteins to assemble the phage nucleus. The phage nucleus not only serves crucial roles in facilitating phage replication and maturation processes but also in protecting the phage genome from bacterial defense mechanisms^29–35^, highlighting their superior antibacterial properties. Thus, we further sought to investigate the genome organization and pinpoint the genes encoding for the defining structures of the nucleus-forming phages^29^. We performed genome comparisons of our phages with the closely-related vibriophages and the well-studied nucleus-forming phage, *Pseudomonas* phage 201Phi2-1^30^. The result demonstrated that despite the low intergenomic similarity score (42.8%) between phages phiKT1019 and phiKT1028, their genome organizations were quite similar and the genes were mostly organized in the same direction (Fig. 4c). However, most regions in their genomes were differently organized compared to those of their closely related phages: vibriophages Aphrodite1 and pTD1 (Fig. 4c). ChmA, which is a major component of the phage nucleus^34^, was present in all of the analyzed phage genomes, including ORF105, ORF71, ORF65, ORF203, ORF180, and ORF65 for *Pseudomonas* phage 201Phi2-1, vibriophages BONAISHI, Aphrodite1, phiKT1019, phiKT1028, and pTD1 (Fig. 4c; pink), supporting the classification of phiKT1019 and phiKT1028 in the *Chimalliviridae* family by the phylogenetic tree. However, the *phuZ* gene, which encodes phage tubulin filaments that orchestrate the process of the phage lytic cycle^36–38^, appeared not much conserved in the vibriophages. Unlike *Pseudomonas* phage 201Phi2-1 (ORF59), vibriophages BONAISHI (ORF59), Aphrodite1 (ORF56), and phiKT1019 (ORF192), the genomes of vibriophages pTD1 and phiKT1028 did not contain the *phuZ* gene (Fig. 4c; yellow), suggesting variations in temporally subcellular regulation for the replication of vibriophages.

To assure that our phages phiKT1019 and phiKT1028 are nucleus-forming vibriophages, we further confirmed experimentally whether the phage nucleus assembles during their infection. We first constructed a fusion of superfolder GFP (sfGFP) to the ChmA proteins (ORF203 for phiKT1019 and ORF180 for phiKT1028) for labeling the wildtype protein and employed fluorescence microscopy to observe the formation of the phage nucleus during infection. In uninfected wildtype cells (Figure S2a; upper panels), the nucleoid of *V. parahaemolyticus* (KT1018B used as a bacterial host; for more genomic data, see Figure S3) formed an irregularly shaped nucleoid that distributed throughout the cells. Moreover, the ChmA proteins of both vibriophages expressed in uninfected cells diffused throughout the cells and did not assemble any specific structures similar to sfGFP control (Fig. 4d and S2b, green). During infection by the vibriophages phiKT1019 and phiKT1028 at 60 min post-infection (mpi), a bright DAPI-staining DNA blob appeared close to the midcell in phiKT1019-infected cells and at the cell center in phiKT1028-infected cells (Figure S2a; middle and bottom panels). The blob of the phage genome is enclosed inside sfGFP-tagged ChmA homologs, which assemble into a ring structure as seen in the Z-cross section (Fig. 4e; green and blue or grey), similar to those previously identified phage nucleus structures^27,30,32,35,39^. No extranuclear DAPI staining due to the host genomic DNA was observed in the cells (Fig. 4e; blue or grey), suggesting that these vibriophages encode some nucleases to destroy the host chromosome during infections. Taken together, our jumbophages phiKT1019 and phiKT1028, as we redesignated them to “Eric” and “Ariel”, respectively, are new members in the *Chimalliviridae *family and are vibriophages that assemble the nucleus-like structure during their lytic life cycle.

### The nucleus-forming vibriophage cocktail prolongs the regrowth of VP_AHPND_ and reduces shrimp mortality in the presence of VP_AHPND_

**Figure 5:**
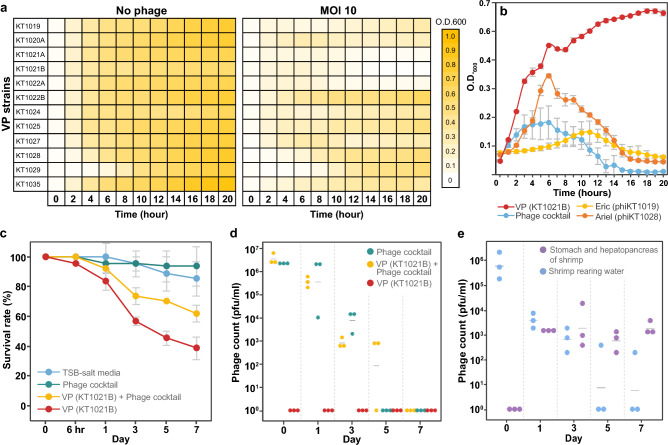
The efficiency of the phage in vitro and in vivo*.* (**a**) Heatmap of growth inhibition of 12 *V. parahaemolyticus* strains in the presence of the phage cocktail at MOI_input_ 10 (left columns) and MOI_input_ 0 (right column; control) throughout 20 h. The O.D. values range from 0 (white) to 1 (yellow). (**b**) Bacterial growth of *V. parahaemolyticus* AHPND strain KT1021B in the absence of phages, in the presence of individual phage (Eric or Ariel at MOI_input_ 10), and in the presence of the cocktail (MOI_input_ 10) during 20 h of incubation. (**c**) The prophylactic performance of the phage cocktail against the infection of *V. parahaemolyticus* AHPND strain KT1021B throughout 7 days and (**d**) the stability of phages in the environmental tanks. The experimental groups were set up as following; TSB-salt media alone (blue), the phage cocktail alone (green), challenge with VP_AHPND_ in the presence of the phage cocktail (yellow), and challenge with VP_AHPND_ (red). (**e**) Colonization of phages in the digestive tract of shrimp. Phage numbers were evaluated in the stomach and hepatopancreas of shrimp (purple) and shrimp rearing water (blue) throughout the 7-day treatment, showing that the phages were retained within the shrimp tract, while becoming not infective in the shrimp rearing water. The experiment was carried out triplicate and the data represent mean ± standard deviation.

With the aim to formulate a cocktail of phages “Eric” and “Ariel” to tackle the pathogenic VP_AHPND_ that currently spreads and causes mass mortality in aquaculture (Table S2), we selected the strains of VP_AHPND_ from the collection of SAAHRDC that appeared to be sensitive to both of our vibriophages (Table 1) and investigated the killing profile of the cocktail against these sensitive isolates (12 isolates). However, due to the difficulty of estimating the target pathogen quantity, as described by Chen et al., (2019), that results in an inaccuracy of the actual optimal MOI, we formulated a phage cocktail containing both phages Eric and Ariel; each at a multiplicity of infection (MOI_input_) of 5, resulting in a total MOI_input_ of 10 relative to the starting bacterial cell number^25^. The in vitro killing profile revealed that the phage cocktail could prolong the regrowth of all tested VP_AHPND_ isolates; however, some bacterial isolates recovered and resumed to grow, resulting in a slight increase in cell density at 20 h of incubation (Fig. 5a). Among the 12 tested isolates, the cocktail efficiently suppressed the bacterial regrowth of 3 VP_AHPND_ isolates throughout 20 h, including strains 1021A, 1021B, and 1022A, with the most prevalent outcome on VP_AHPND_ strain 1021B (Fig. 5a), indicating the potency of the cocktail against these isolates. The use of the combination of phages (Eric + Ariel) against the strain 1021B was proven to be more effective than the use of single phage alone. At the same MOI_input_, the cocktail initially suppressed the bacterial growth as early as 6 h of treatment, followed by the decrease in bacterial cell density to the baseline at 15 h. Moreover, the regrowth of bacteria was not observed during the treatment of the cocktail until 20 h of incubation (Fig. 5b). Together with the high pathogenicity of VP_AHPND_ strain 1021B, which causes 95% mortality in Pacific white shrimp (*Penaeus vannamei*) (Table S2), this strain was thus selected as a representative of AHPND strains to further validate the performance of the cocktail against VP_AHPND_ infection in vivo*.*

We first focused on two main aspects of the cocktail, including the prophylaxis efficiency against VP_AHPND_ and the phage stability in the real environmental tanks. According to the prophylactic activity of the cocktail against VP_AHPND_, we evaluated the survival rate of shrimp over a window of 7 days in the following conditions: media control, challenge with VP_AHPND_ in the presence of the phage cocktail, and challenge with VP_AHPND_. With the concern of phage-mediated toxicity, we also included a condition of the phage cocktail alone. The result revealed that, with a challenge with VP_AHPND_, almost 20% of shrimp died upon the challenge at day 1, followed by a sharp increase in mortality leading to only 40% surviving shrimp at day 7 (Fig. 5c; red line). The presence of the phage cocktail can prolong and reduce shrimp mortality. The phage cocktail retained the survival rate of infected shrimp at a similar level compared to the media control at day 1 (Fig. 5c; yellow and blue lines). However, since the presence of the vibriophages sharply declined at day 5, in the cultured conditions (Fig. 5d), the cocktail failed to rescue the infected shrimp at the following period, resulting in a 60% survival rate at day 7 (Fig. 5c; yellow line). The survival rate of shrimp in the presence of the cocktail was 20% higher than that in the absence of the cocktail, suggesting the activity of the cocktail in preventing the animals from infection (Fig. 5c; yellow and red lines). The shrimp survival rates in the conditions of the media control and the phage cocktail alone were at a comparable level (Fig. 5c; blue and green lines), suggesting undetected side effects from the phage cocktail.

Since we observed the low stability of our vibriophages in shrimp-rearing water, we questioned whether the phages would be able to colonize the open digestive tract of shrimp. To evaluate the colonization of phages in the shrimp digestive system, we dissected the stomach and hepatopancreas of shrimp in the experiment and determined the phage titer for at least 7 days in comparison to the phage titer in shrimp rearing water (Fig. 5e). At day 0, the phage titer in shrimp-rearing water was approximately 10^5^–10^6^ pfu/ml, which was similar to the concentration of the phage cocktail initially added to the water. The number of phages, again, dropped substantially to around 10^3^ pfu/ml at day 3 and was almost completely diminished (less than 10 pfu/ml) at day 5 and day 7 (Fig. 5e; blue), suggesting the vibriophages’ low tolerance in the rearing water. Surprisingly, the number of phages in the digestive tract of shrimp increased significantly from an undetectable level at day 0 to approximately 10^3^ pfu/ml at day 1 (Fig. 5e; purple). Moreover, the phage numbers in the digestive tract remained relatively constant throughout the 7-day experimental period (Fig. 5e; purple). Taken together, this phage cocktail, which is derived from the nucleus-forming vibriophages Eric and Ariel, is effective in preventing shrimp from VP_AHPND_ infection and is capable of colonizing the digestive tract of shrimp.

## Discussion

The spread of pathogenic VP_AHPND_ contributes to global losses in the shrimp farming industry^5,7^. Unfortunately, the use of antibiotics, a conventional approach, has limited success due to multidrug-resistant (MDR) variants^40^. Bacteriophage therapy, the use of prokaryotic viruses targeting bacteria, is now considered a future alternative strategy to combat pathogenic infection and has been proven by numerous successful cases worldwide^23,41^. Here, we present an integrated workflow starting from high-throughput phage screening, followed by characterization of phage kinetics and genomic data, and the application of phage as a biocontrol. With our high-throughput phage screening adapted from the previous published HiTS method^26^, we can rapidly identify many potential natural resources that contain phages targeting the bacterial isolates of interest. Since the screening of a variety of bacterial isolates and various natural samples was conducted in a checkerboard manner, this screening generally increases the possibility of obtaining diverse phages in a single screen, thus avoiding the laborious work of traditional phage screening. However, with this screening, it is worth mentioning here that the elementary criteria, including clear plaque morphology and host spectrum, are still needed for the selection of phage candidates appropriate for use. In this screening of 98 resources against 28 pathogenic VP_AHPND_, we obtained 12 potential resources that contain at least 14 different phages. Among these, phages phiKT1019 (Eric) and phiKT1028 (Ariel) that displayed the broadest host spectrum were selected as candidates due to our primary selection criteria described above.

Selection of phages for biocontrol and therapy is a crucial step, and selection of inappropriate phages could result in ineffective treatment^42^. Lytic or virulent phages are highly recommended for the use in bacterial infection treatment and prophylaxis to avoid horizontal gene transfer that would make the bacteria much more threatening, as in the case of temperate phages^43^. Here, we have carefully determined that phages, Eric and Ariel, are safe and appropriate for use in biocontrol. Their genomes did not contain any toxin genes and antibiotic-resistance genes. In addition, the absence of lysogenic-related genes as of the genomic data and the lysogeny assay in phage-resistant isolates experimentally support that the phages are indeed lytic phages. However, using these phages might have some drawbacks due to the relatively low adsorption rate to the bacterial host and the production of low progeny after the lytic cycle compared to other phages^44^. This is unsurprising as it has been known that giant phages that harbor large genomes would require more replication period and highly organized replication that would result in the low production of phage progeny^45^.

Phages Eric and Ariel are nucleus-forming vibriophages that belong to the *Chimalliviridae* family due to the presence of the *chmA* gene in the genome. The *chmA* gene, as one of the defining structures for nuclear-based replication, is conserved and present across diverse nucleus-forming jumbophages, including *Erwinia*, *Ralstonia*, *Escherichia*, *Salmonella*, *Pseudomonas*, and *Vibrio* phages^27,29^. During infection by the phages in this family, they encode the ChmA protein along with other accessory proteins to assemble a proteinaceous shell to enclose the phage genome and partition proteins according to their functions, allowing enzymes involved in DNA replication and RNA transcription inside while excluding other enzymes involved in protein translation and nucleotide synthesis on the outside^30,34^. This structure is referred to as the phage nucleus because of its biological functions that resemble those of the eukaryotic cell nucleus^32,46^. In addition to serving as the nucleus during phage replication, in an evolutionary arms race between them and their bacteria host, the phage nucleus also protects the phage genome from host defense mechanisms that target DNA, such as Clustered Regularly Interspaced Short Palindromic Repeats (CRISPRs) and Cas enzymes, and restriction-modification enzymes, thus overcoming the host defense^33,35^. Therefore, the ability of vibriophages Eric and Ariel to assemble the phage nucleus to protect their genome from the DNA-targeting defenses of the bacteria is greatly advantageous from an application standpoint, as they would be more tolerant in long-term application than phages in other families.

PhuZ-based filaments, previously thought to be another defining structure for nuclear-based replication as they spatially and temporally organize the phage nucleus position and capsid maturation, are not obligatory conserved in the *Chimalliviridae* family^27,29^. Previous studies have revealed that disruption of the dynamic stability of PhuZ filaments leads to the mispositioning of the phage nucleus and a failure to deliver procapsids to the nucleus for DNA encapsidation, resulting in decreased phage progeny^36–38,47^. Our study demonstrated that phage Ariel has a slightly higher number of phage progenies than phage Eric, which assembles the PhuZ-based filaments. This suggests that nucleus-forming phages that lack the phuZ gene in their genome might exploit alternative subcellular organizations to achieve optimal phage progeny production. Other organizations that might complement the absence of PhuZ filaments require further investigation. Our recent research has implicated PhuZ as a factor contributing to virogenesis incompatibility for viral speciation, as homologs of PhuZ from distinct phages can co-assemble but produce non-functional static hybrid filaments^48^. Therefore, infection of a single bacterial cell with 2 nucleus-forming phages encoding PhuZ homologs that would occur in the phage cocktail might hamper the effectiveness of the phage combination. Fortunately, since one of the phages in our combination lacks a PhuZ homolog, the viral incompatibility mediated by the non-functional hybrid filaments would not occur, rendering the combination of our vibriophages compatible and effective for use. Since the combination of phages, which possess different cellular organizations, enhances the overall efficiency of the cocktail^49^, insights into host-phage interaction at the cellular level during co-infection of these phages will be necessary to warrant the compatibility of phages in the future.

According to the appropriate properties of the candidates, phages Eric and Ariel, described above, the phage cocktail was formulated and was shown to produce desirable outcomes in both in vitro and in vivo studies. The cocktail completely suppressed the regrowth of three highly pathogenic strains of VP_AHPND_, previously isolated from infected shrimp in local farms. In addition, the use of a cocktail containing polyvalent phages was more effective than the use of monovalent phages in reducing the bacterial density, ensuring the advantage of the phage cocktail. The maximum efficiency of the phage cocktail (VP_AHPND_ + cocktail) appears to be maintained within the first 24 h, as evidenced by shrimp survival rates, which are comparable to those observed in the absence of VP_AHPND_ but are higher than those observed in the presence of VP_AHPND_ alone (Fig. 5c). However, the activity of the cocktail in the condition (VP_AHPND_ + cocktail) was reduced thereafter as the cumulative mortality reached 40% at day 7. The cumulative mortality is still lower than that of the conditions in the presence of VP_AHPND_ alone at day 7, indicating the prophylactic effect of the cocktail. This is partly due to the low phage stability in the cultured tanks, which is likely influenced by unknown environmental factors (Fig. 5d and 5e). Further development for maximizing phage stability in environments will be needed to enhance the phage cocktail efficiency. Even though the phages are not tolerant of the environment, they are capable of colonizing the open digestive tract of shrimp and being retained for at least a week (Fig. 5e). It has been reported in moribund shrimps that phages are able to penetrate into the hepatopancreas through hemolymph^50^, and the phage particles in the digestive tract are likely maintained within the mucosal surface of the shrimp stomach by the interaction between Immunoglobulin (Ig)-like domains on the phage capsid and mucin peptidoglycans^51,52^. In this study, the organs in shrimp where phages colonize are still ambiguous, as phages were titered from the mixture of the ground digestive tract. This will further form the basis for an examination of the process of colonization by phages and specific organs where phages interact. Moreover, since shrimp immersion in seawater containing a phage cocktail appears to allow the phages to colonize in the gut, but at low quantities (~ 10^3^ pfu/ml), feeding shrimps a diet supplemented with the cocktail may contribute to more phage particles adhering within the tract and would be a further application worth pursuing. This research on a phage cocktail derived from nucleus-forming vibriophages still has room for advancement and would soon provide a potential preventive strategy against AHPND infection.

## Material and methods

### Bacterial strains and growth

To make an overnight culture, *V. parahaemolyticus* (Table 1) were grown on tryptic soy agar plates supplemented with 1.5% NaCl (TSA-salt) at 30 °C and then inoculated into tryptic soy broth supplemented with 1.5% NaCl (TSB-salt) at 30 °C, for at least 16 h, vigorously shaking at 250 rpm. To make a day culture, the overnight culture was inoculated 1:100 into TSB-salt until the early exponential growth (the optical density at 600 nm = 0.2 or 0.4) by shaking at 250 rpm, 30 °C. The day culture was used in following experiments: phage adsorption, one-step growth curve, single cell-level study, determination of shrimp mortality in VP_AHPND_ strains, phage cocktail formulation in vitro and the efficiency of phage cocktail against AHPND-infected shrimp in vivo*.*

This work has been reviewed and approved by Chulalongkorn University-Institutional Biosafety Committee (CU-IBC) in accordance with the levels of risk in pathogens and animal toxins, listed in the Risk Group of Pathogen and Animal Toxin (2017) published by the Department of Medical Sciences (Ministry of Public Health), the Pathogen and Animal Toxin Act (2015) and Biosafety Guidelines for Modern Biotechnology BIOTEC (2016), with approval number: SC CU-IBC-028/2020.

### Vibriophage screening

According to the phage isolation method of Olsen et al.^26^, we employed the high-throughput screening (HiTS) method with modifications. To easily handle a large number of seawater samples, we adjust smaller final volumes per well and use chloroform to eliminate bacteria instead of filtration. On top of that, we allowed the phage to propagate for 2 days, which was longer than the HiTS method. Our method is able to apply for isolating various phages from other environmental samples by adjusting the host medium, buffers and incubation conditions. To this, our developed method was roughly explained into 4 steps as follows; (I) Sample preparation, (II) Phage propagation, (III) Bacterial elimination and (IV) Spot test, as illustrated in Fig. 1.

### Phage purification and propagation, plaque morphology, and phage structure

In order to purify and propagate phages, a double-layer agar (DLA) technique was used as described by Thammatinna et al.^53^. Briefly, for phage purification, the mixture of *V. parahaemolyticus* overnight culture (100 µl) and phage lysate (100 µl) was added into 5 ml semi-solid TSB-salt agar (0.35% agar) and then immediately poured on TSB-salt agar followed by incubation at 30 °C for 24 h. A single plaque was picked from the double-layer agar plate and suspended in 100 µl of SM buffer (100 mM NaCl, 8 mM MgSO_4_, 50 mM Tris-HCl pH 7.4, 0.002 w/v gelatin). To ensure a single phage, this step was repeated at least three times.

For phage propagation, phage was amplified by a double-layer agar technique plate as described above. After incubation, 5 ml of SM buffer was added to the web lysis plate followed by incubation at room temperature for 5 h allowing phage to release into the buffer. Then, the phage lysate was collected by centrifugation at 9000 rpm for 15 min and the supernatant was filtrated through a 0.45 µm filter. Phage lysate was maintained in SM buffer and subsequently stored at 4 °C until use.

To investigate phage morphology, a transmission electron microscope (TEM) was performed. The clear plaque on the lawn of the parental host was picked and resuspended in SM buffer. After centrifugation at 9000 rpm for 2 min, the supernatant was dropped onto copper grids and negatively stained with 2% w/v uranyl acetate (pH4.5). The phage morphology was observed by a Hitachi HT7700 at 80–100 keV.

### Host range determination

The host spectrum of the isolated phage against 31 strains of *V. parahaemolyticus* (Table 1) was determined by using the spot test*.* Briefly, 5 µl of phage lysate was dropped onto the soft-agar plate surface, which was prepared by mixing 100 µl of bacterial overnight culture with 5 ml of the 0.35% semi-solid agar and overloading onto the TSB-salt plate. The formation of the translucent spot on the bacterial lawn was recorded after 16 h of incubation at 30 °C.

### Phage adsorption and one-step growth curve

The evaluation of phage adsorption and the one-step growth curve was performed as described in a previous study by Thammatinna et al.^53^. *V. parahaemolyticus* was cultured in TSB-salt as mentioned previously. For the adsorption assay, the bacterial culture at an optical density at 600 nm (O.D._600_) of 0.4 indicating ~ 4 × 10^8^ cfu/ml was infected with phage at a multiplicity of infection (MOI_input_) of 0.01 or 4 × 10^6^ pfu/ml in a static condition at 30 °C. The 100 µl of samples were immediately taken to 900 µl of SM buffer at the following time points: 0, 1, 2, 5, 7.5, 10, 15, 20, 25, and 30 min, and then they were centrifuged at 12,000 rpm at 4 °C for 1 min. The non-adsorbed free phage in the supernatant at each time point was evaluated using the double-layer agar method.

A one-step growth curve analysis was performed to determine the latent period and burst size of the isolated phages. Mid-log phase growth *V. parahaemolyticus* cultures were infected with phage as described in the above condition. Phage particles were allowed to attach to their host for 15 min at 30 °C followed by removing the non-adsorbed free phage by centrifugation at 10,000 rpm for 5 min. The infected cells were suspended in 10 ml of TSB-salt media and incubated in a static condition at 30 °C for 2 h. At the desired time points, the bacterial cells were then removed by centrifugation at 9000 RPM for 2 min, and the supernatant was collected to evaluate phage number using the double-layer agar method. The calculation of the burst size is provided below.

$${\text{Burst}}\;{\text{size}}\left( {{\text{particles}}\;{\text{per}}\;{\text{cell}}} \right){ = }\frac{{{\text{Average}}\;{\text{phage}}\;{\text{number}}\;{\text{at}}\;{\text{the}}\;{\text{plateau}}\;{\text{phase }} - {\text{Average}}\;{\text{phage}}\;{\text{number}}\;{\text{at}}\;{\text{the}}\;{\text{latent}}\;{\text{phase}}}}{{{\text{The}}\;{\text{initial}}\;{\text{phage}}\;{\text{yield}}}}$$

### Phage tolerance: pH and temperature

The stability of the isolated phages was examined at different pHs and temperatures. To evaluate the pH stability, 50 µl of each phage lysate containing around 1 × 10^6^ pfu/ml was incubated with 450 µl of SM Buffer with the following pH values; 2, 4, 6, 7.4, 8, 10, and 12 at 30 °C for 1 h. For thermal stability of the phage, 50 µl of 1 × 10^6^ pfu/ml phage lysate was kept at different temperatures for 1 h as follows: 4, 35, 30, 37, 40, 50, 60, and 70 °C. The survival phages were titered by using a spot test, as described previously.

### Lysogeny test

To determine whether phage is temperate phage, a phage-resistant strain containing prophage in its genomes was investigated by induction with mitomycin C. Firstly, a phage-resistant strain was isolated from the parental host lawn containing a high titer of phages. Secondly, to confirm the resistant strains, high-titer phage lysate was dropped onto the cross-streaks of the isolated strains compared with the wild-type strain. Lastly, the wild-type strain was grown in semi-solid TSB-salt agar supplement with 0.05% mitomycin C and then resistant-strain was stabbed using a sterile toothpick. The DLA plate was incubated at 30 °C for at least 16 h. Positive clear zone indicated the presence of prophage in *V. parahaemolyticus* genome. The phage lysate was used as positive zone. The triplicate experiment was conducted.

To further confirm the presence/absence of prophage in the phage-resistant genomes, conserved genes including capsid and *recA* genes of each phage were detected in the genome by PCR method. Briefly, genomic DNA of the phage-resistant strains was extracted by using the colony boiling method. The colony of resistant bacteria was picked, transferred to 100 µl deionized water, boiled at 100˚C for 10 min, and placed on ice for 15 min. The oligonucleotide primers of both conserved genes of each phage were designed including capsid gene of phiKT1019; forward: 5′-GCTGCA GGAGGCAGCCAAAAAATGAAAAGAAATATCGTAGCTC-3′ and reverse: 5′-GCCTGCAGGTCG ACTCTAGACTATGGCAACTTACCTGCATC-3′, *recA* gene of phiKT1019; forward: 5′-GCTGCA GGAGGCAGCCAAAAAATGTCCACCGCTTTTAATTTC -3′ and reverse: 5′-GCCTGCAGGTCGACTCTAGATTACTTTTTCTTAGGACGACC-3′, capsid gene of phiKT1028; forward: 5′-GCTGCAGGAGGCAGCCAAAAAATGAAAAGAAATATCGTAGCTC-3′ and reverse: 5′-GCCTGCAGGTCGACTCTAGATTATGGTAACTGACCAGTTTC-3′, and *recA* gene of phiKT1028; 5′-GCTGCAGGAGGCAGCCAAAAAATGTCAATTCCGAATTTCG-3′ and reverse: 5′-GCCTGCAGGTCGACTCTAGATTATTTTTTTGCAGGACGTC-3′. PCR mixture contained 1X PCR buffer (0.25 mM dNTP, 1.5 mM MgCl_2_), 1 µM of each forward and reverse primer, 1 U Taq DNA polymerase (Thermo scientific), and 5 µl of DNA template in a total volume of 25 µl. The reaction was carried out in Bio-Rad T100 Thermal cycler as follows: 94 °C for 1 min; 35 cycles of 30 s at 94 °C, 45 s at 55 °C, and 1 min at 72 °C; 10 min at 72 °C. The expected size of the capsid and *recA* genes were 2195 bp and 1434 bp for phiKT1019 and 2180 bp and 1422 bp for phiKT1028. The PCR products were investigated by agarose gel electrophoresis in 1x TBE buffer (0.04 M Tris-acetate, 0.001 M EDTA [pH 8.0]) and visualized by UV transilluminator.

### Phage DNA extraction, phage genome sequencing and genome analysis

Phage particles were precipitated with a 20% phage precipitant solution (30% w/v PEG-8000, 3.3 M NaCl, and sterile distilled water) at 4 °C overnight and centrifuged at 10,000 rpm for 30 min. To degrade bacterial DNA and RNA, the pellet was resuspended in 1 × DNaseI buffer and incubated with 5U DNaseI and 25 µg RNaseA at 37 °C for 2 h. The nuclease activity was inhibited by adding 25 mM EDTA, and after that, phage capsids were digested with 0.5% SDS and 25 µg proteinase K at 60 °C for 2 h. Lastly, phage genomic DNA was extracted using the conventional phenol–chloroform method.

Whole-genome libraries were carried out on the Illumina HiSeq platform and qualified by FastQC^54^. The contaminated host DNA fragments were filtered out by using Bowtie2^55^ and Samtools^56^ against *V. parahaemolyticus* genomes. Lastly, the filtered reads of each phage were assembled as a single contig by SPADES^57^.

The formation of the phage genome (in linear or circular form) was determined by PhageTerm^58^. Open reading frames (ORFs) were predicted by GeneMark^59^ and assigned by the algorithms of BLASTp^60^ and PSI-BLAST^60^ (cutoff e-value < 10^−3^) against the following databases: non-redundant (nr) protein databases of NCBI, InterPro 75.0^61^, NCBI conserved domain^62^ and ACLAME^63^. The predicted genes were also automatically confirmed by the RAST server annotation service^64^ and PHASTER^65^. The presence of antimicrobial resistance genes and putative toxins was determined using BLAST against RESFINDER and VirulenceFinder^66^. The tRNAScanSE^67^ was used to examine the putative transfer RNA (tRNA). The genome map was produced by the DNA plotter tool of Artemis software^68^.

A phylogenetic relationship was constructed by the VICTOR Virus Classification web tool^69^, Tree Building Online Resource, Molecular Evolutionary Genetics Analysis (MEGA) version 10.0^70^, and Figtree program against complete phage genomes that were selected from the GenBank database. The intergenomic similarity and comparative genome of selected phage genomes were conducted by the VIRIDIC web tool^28^ and EasyFig 2.1^71^, respectively.

### Bacterial whole genome sequencing, genome assembly and annotation

The DNA of *V.parahaemolyticus* KT1018B (or J42) was extracted using a bacterial DNA extraction kit (Vivantis). Its quality was evaluated by Nanodrop and agarose gel electrophoresis with A_260_/A_280_ > 1.90 and no degraded pattern. The paired-end 2 × 150-bp sequencing library was prepared, and the sequencing was performed using the DNBSeq platform (BGI Tech Solutions (Hongkong) Co., Ltd.). The sequencing output was evaluated for quality with Q20 > 95%. The raw reads were removed from low quality sequences and remaining adapters by SOAPnuke software^72^.

Cleaned reads were assembled and annotated using NCBI Read Assembly and Annotation Pipeline Tool v0.5.3 (RAPT, https://www.ncbi.nlm.nih.ov/rapt). In brief, the pipeline consists of three major parts: genome assembly using SKESA^73^, taxonomic assignment inferred from the average nucleotide identity (ANI) matrix and annotation using the NCBI Prokaryotic Genome Annotation Pipeline (PGAP)^74^, and production of an annotated genome of quality comparable to RefSeq. Associated gene ontology (GO), cluster of orthologous groups (COG), and KEGG pathway terms were then identified using EggNOG-mapper v2.1.9^75^. Assembled contigs and associated features were visualized as a circos plot using the Proksee (https://proksee.ca) web application.

### Plasmid constructions and bacterial transformation

The genes of interest were amplified from high-titer phage lysate using polymerase chain reaction amplification using specific primers as listed in Table S3. After that, the recombinant plasmids were constructed by insertion of each amplicon into the linearized backbone of pBAD33 using the NEBuilder HiFi DNA Assembly Cloning Kit. The constructs listed in Table S4 and S5 were transformed into electrocompetent *Escherichia coli* DH5ɑ by electroporation method. The transformants containing recombinant plasmids were selected by Luria–Bertani (LB) plates supplemented with 30 µg/ml chloramphenicol and confirmed by colony PCR. The correctness of the recombinant plasmid was evaluated by DNA sequencing. The constructs with the correct sequence were extracted from *E.coli* DH5ɑ followed by transformation into the original strain of *V. parahaemolyticus* as described in detail in Table S6. The transformants were selected by LB plate supplemented with 5 µg/ml chloramphenicol.

### Single cell-level study

The original strain of *V. parahaemolyticus* (KT1018B) was selected to study the mechanism of pre-killing (MOK) of the isolated phages. The bacterial strain was grown in TSB-salt media with vigorous shaking at 250 rpm 30 °C. After O.D._600_ of the culture reached 0.4, 1 ml bacterial culture was treated with phage lysate at MOI_input_ 1. The bacterial cells were allowed to infect with phage particles for 1 h at 30 °C in the static condition. Bacterial culture with mock lysate was set as a negative control. To this, the cells were fixed for 1 min with the final concentration of 2% paraformaldehyde and 2% glutaraldehyde followed by centrifugation at 9000 rpm for 1 min. The cells were suspended in 500 µl 1xPBS buffer containing 1 µg/ml FM4-64 for membrane staining and 2 µg/ml DAPI for DNA staining. After 1-min staining, stained cells were pelleted and dropped onto an agarose pad (1.2% agarose in 20% TSB-salt media), prepared on a concavity slide. The DeltaVision Spectris Deconvolution microscope was performed to visualize the cells with consistent imaging parameters. The images were further processed by the deconvolution algorithm in DeltaVision SoftWoRx Image Analysis Program.

Further insights into the vibriophage-infected cell were provided by visualizing the infected cells tagging ChmA gene of the vibriophages. The experiment was performed according to the above experiment with minor modifications. Briefly, *V. parahaemolyticus* colony was inoculated in TSB-salt media containing 5 µg/ml chloramphenicol at 250 rpm 30 °C for 16 h. The saturated culture was diluted 100-fold in TSB-salt media without the selective antibiotic and incubated at 250 rpm 30 °C. After the early exponential growth (O.D._600_ of 0.1) was observed, protein expression was induced by 0.2% l-arabinose followed by incubating at 250 rpm 30 °C. Bacterial cells were then infected with the phages with MOI_input_ 1 when O.D._600_ of the culture reached 0.4. After this, the experiment was performed as described above.

### The formulation of phage cocktail to suppress VP_AHPND_ in vitro

In order to determine the killing efficiency of the phage cocktail formulation against *VP*_*AHPND*_ strains (Table 1) at MOI_input_ of 10, 15 selected bacterial strains were included to test the inhibition effectiveness of the cocktail. The bacteria were grown in TSB-salt to reach O.D._600_ of 0.4 which was equal to approximately 4 × 10^8^ cfu/ml. After that 250 µl of each bacterial strain was transferred to the 96-well plate containing phage cocktail. The phage cocktail formulation involved the combination of each phage at a 1:1 ratio, with concentrations of 2 × 10^9^ pfu/ml for each phage, resulting in a final concentration of 4 × 10^9^ pfu/ml or MOI_input_ of 10. The bacterial cultures without phage cocktail treatment were set up as positive control. The cultures were incubated at 30 °C for 20 h and measured the value of O.D._600_ at 1-h intervals. The experiment was conducted in triplication.

The bacterial strain with the highest suppression (VP_AHPND_ strain KT1021B) was selected to validate the bacterial suppression performance of the individual phage candidates (MOI_input_ of 10) and phage cocktail (MOI_input_ of 10). Phage kinetic was performed by measuring the value of O.D._600_ as described above.

### Determination of shrimp mortality in VP_AHPND_ strains

The mortality of Pacific white shrimps (*P. vannamei*) with an average weight of 5 g against *V. parahaemolyticus* strains as listed in Table S2 was evaluated over 2 weeks. The 5 colonies of each *V. parahaemolyticus* strain were picked and resuspended in normal saline to reach O.D._640_ of 0.1. Thirty microliters of bacteria suspension were added into 300 ml of TSB supplemented with 2% NaCl followed by incubation at 30 °C, for at least 14 h with shaking at 150 rpm. Then, the overnight culture was adjusted in TSB supplemented with 1.5% NaCl to reach O.D._640_ of 1 (1 × 10^9^ cfu/ml). For immersion challenge, 30 healthy shrimps were immersed in 20 L of seawater (15 ppt) containing *V. parahaemolyticus* suspension at a final concentration of 8.3 × 10^6^ cfu/ml for 6 h. After immersion, 16 L of shrimp rearing water in tanks was replaced by new seawater. On top of that, shrimp rearing water was changed daily. Shrimp mortality was recorded 3 times a day throughout 14 days. The shrimps without bacterial suspension were served as control group. The experiments were conducted in duplicate.

### The application of cocktail against AHPND-infected Pacific white shrimp in vivo

The therapeutic application of phage therapy in situ against AHPND (*V. parahaemolyticus* strain KT1021B) infected Pacific white shrimps was determined by counting the survival rate of shrimp and phage titer in shrimp rearing water throughout a week. The experimental groups were set up as follows; (I) adding TSB-salt media, (II) treating only phage cocktail, (III) challenging with AHPND and receiving the treatment of phage cocktail and (IV) challenging with AHPND.

Healthy Pacific white shrimp with an average weight of 5 g were obtained from Songkhla Aquatic Animal Health Research and Development Center (SAAHRDC), Department of Fisheries, Thailand for use in the project. During the early 6 h, each experimental group contained 20 healthy shrimps per replicate in 15 L seawater. Group I was supplemented with 25 ml TSB-salt media. Group II was treated with a phage cocktail at a final concentration at around 8.3 × 10^7^ pfu/ml. For experimental group III and IV, shrimps were allowed to infect by immersion with the mid-log phase AHPND culture at a final concentration of 8.3 × 10^6^ cfu/ml whereas group III was also treated with a phage cocktail at 8.3 × 10^7^ pfu/ml (MOI_input_ of 10). After 6 h, 15 L of seawater was added to all groups to finally reach the final volume of 30 L. The survival rate of shrimp was recorded; phage particles in shrimp rearing water were counted by spot test at day 0, 6 h, 1, 3, 5 and 7. To keep phage particles in the systems, seawater in each tank was not allowed to change throughout 7 days but the strainer was used to filter debris. The experiments were carried out in triplicate. Another experiment was set up as described in group II to compare phage particles between colonizing in the stomach and hepatopancreas of shrimp, and shrimp rearing water. The stomach and hepatopancreas of shrimp and shrimp rearing water were collected throughout the 7-day treatment to titer phage particles by using a spot test.

This project with the animal use protocol number 2123016 has been reviewed and approved by the Certification of Institutional Animal Care and Use Committee (IACUC) in accordance with university regulations and policies governing the care and use of laboratory animals. The review has followed guidelines documented in Ethical Principles and Guidelines for the Use of Animals for Scientific Purposes edited by the National Research Council of Thailand.

### Supplementary Information

Supplementary Information.

## Data Availability

Genomes of phage phiKT1019 and phage phiKT1028 have been submitted to the NCBI GenBank database under the accession number MZ622713 and OP123707, respectively. The *V. parahaemolyticus* genome sequence was deposited in the NCBI database under BioProject accession number PRJNA886466, including raw reads under SRA accession number SRR21796858. In addition, the assembled contigs were deposited as Genbank accession number JAOSLV000000000.1.
