# Supplementary material for: Nucleus-forming vibriophage cocktail reduces shrimp mortality in the presence of pathogenic bacteria
Source: Sci Rep. 2023 Oct 19;13:17844. doi: 10.1038/s41598-023-44840-x (PMC10587174; doi:10.1038/s41598-023-44840-x)
Supplement: Supplementary file 1 — Supplementary Information. [file 41598_2023_44840_MOESM1_ESM.docx]

**Nucleus-forming vibriophage cocktail reduces shrimp mortality in the presence of pathogenic bacteria**

**Supplementary information**

**
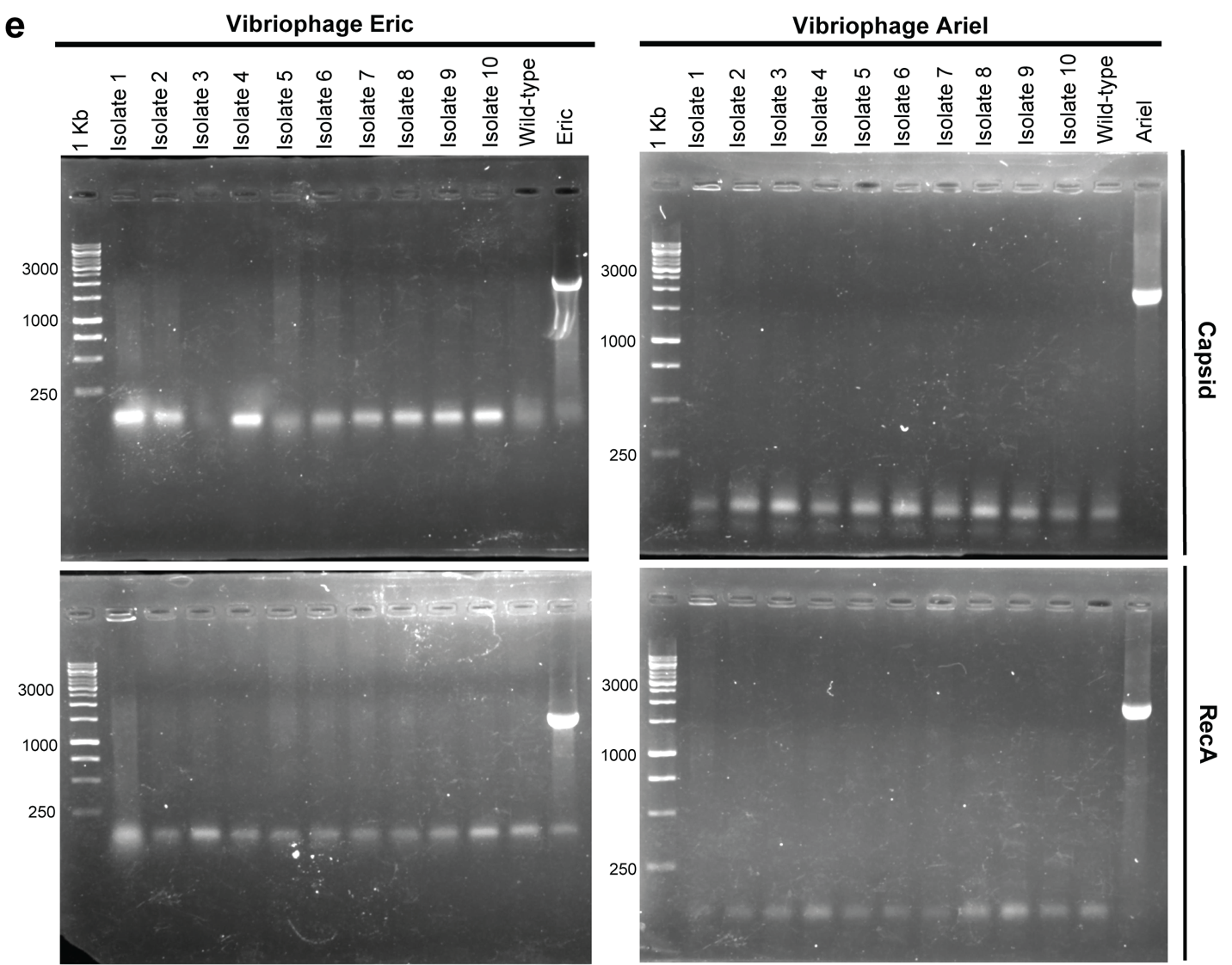
**

**Figure S1** Lysogeny test of phages phiKT1019 and phiKT1028. The bacterial strains resistant to phages phiKT1019 (a, isolates 1- 5) and phiKT1028 (b, isolates 1- 5) were raised and confirmed the resistance ability by a cross-streak test. Arrowheads indicate where a high titer phage was dropped atop the streaks. The lysogeny assay demonstrated that plaques which indicate the presence of prophage in the resistant bacteria were not observed in both resistant bacteria to phages phiKT1019 (c, isolates 1- 5) and phiKT1028 (d, isolates 1- 5) after activation of mitomycin C. Arrowheads indicate clear zones which were the result of cell lysis in the positive control. E. PCR amplification of capsid and RecA genes from the phages Eric and Ariel’s genome that would be potentially integrated in the host genome as a lysogen. Ten phage-resistant isolates raised from each phage were listed as isolate 1-10. Wild-type bacterial host sensitive to the phages was used as the negative control. Phage lysate was used as the positive control.

**
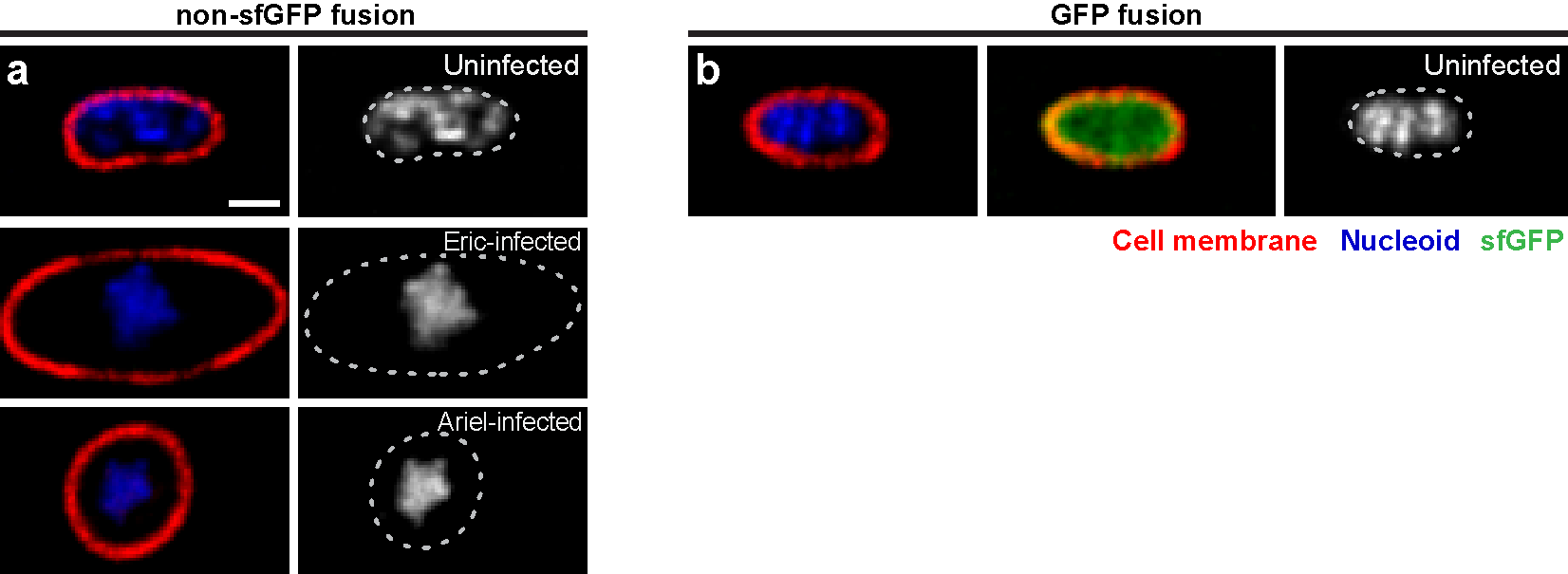
**

**Figure S2** Morphology of uninfected and phage-infected *V. parahaemolyticus* KT1018B. (a) Fluorescence images of wildtype *V. parahaemolyticus* KT1018B in the absence of phage (upper panel), during infection with phage Eric (middle panel), and during infection with phage Ariel (bottom panel). (b) Fluorescence images of uninfected *V. parahaemolyticus* KT1018B expressing sfGFP showing the uniform distribution of sfGFP control (green). DNA was stained with DAPI (blue or grey) and cell membrane was stained with FM4-64 (red). Dashed lines indicate cell borders. Scale bars, 1 micron.

**
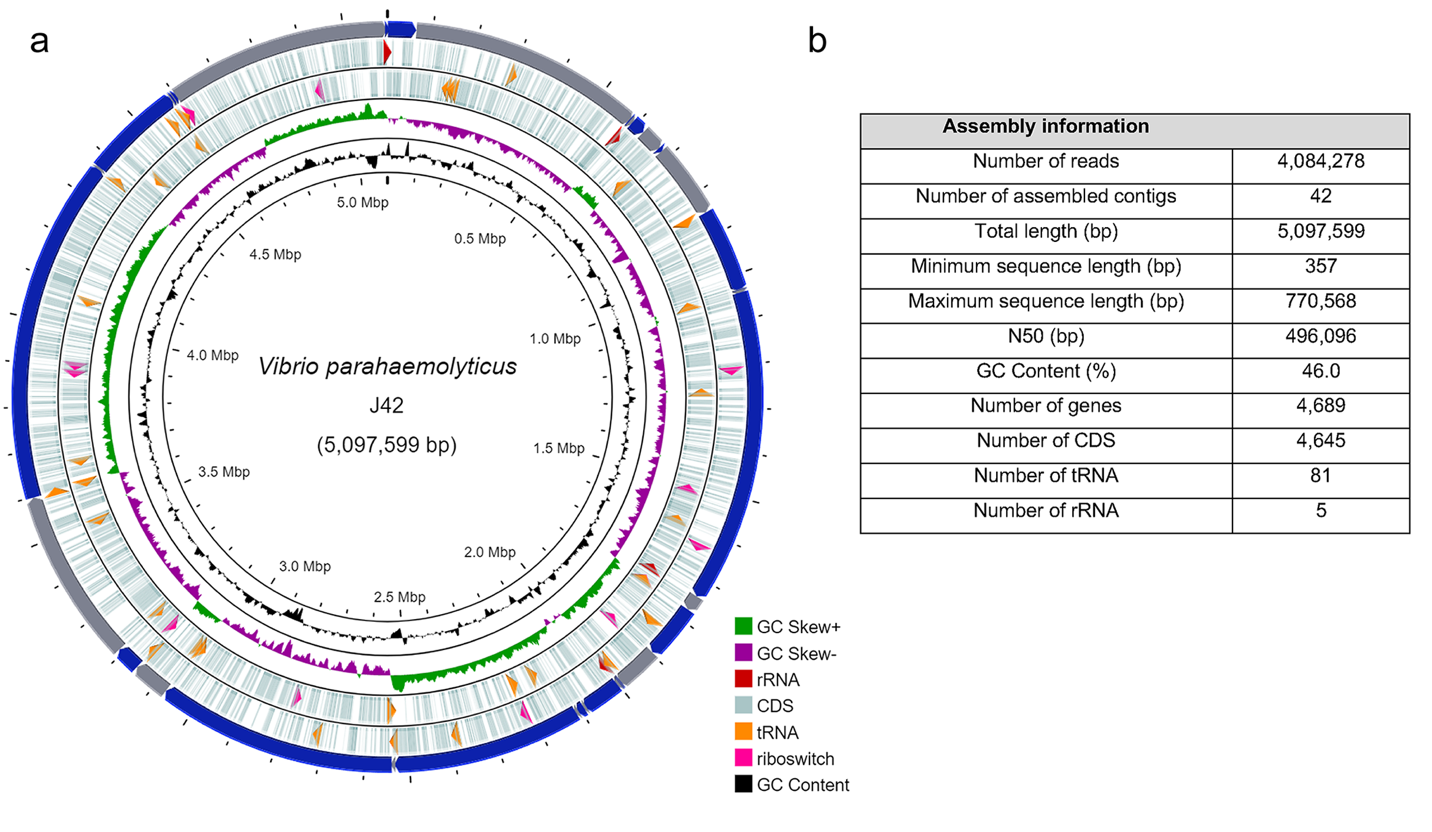
Figure S3** (**a**) Circos plot of *V. parahaemolyticus* KT1018B genome. The draft genome of *V. parahaemolyticus* KT1018B was analyzed by CGViewer Builder via Proksee webserver. Contigs (blue and grey) represent in the outermost with following the distribution of sense and antisense genes in the inner layers, respectively. The red, orange, and pale blue represent rRNA, tRNA, and cds, respectively. The innermost is a distribution of GC content with positive (green) and negative (purple) GC skewness. (**b**) Assembly statistics of *V. parahaemolyticus* KT1018B. The genome is available in the NCBI database under Genbank accession number JAOSLV000000000.1.

**Table S1** The analysis of plaque morphologies of enrichments obtained from the high-throughput screening (HiTS) method for phages*,* with slight modifications (Figure 1). The appearance of plaques was counted as “hit”. Plaque morphologies of enriched phages from seawater samples (Y-axis) versus V. parahaemolyticus strain (X-axis) were recorded as follows: red for clear plaque, orange for turbid plaque, yellow for bull’s eye plaque, and white for no plaque.

**
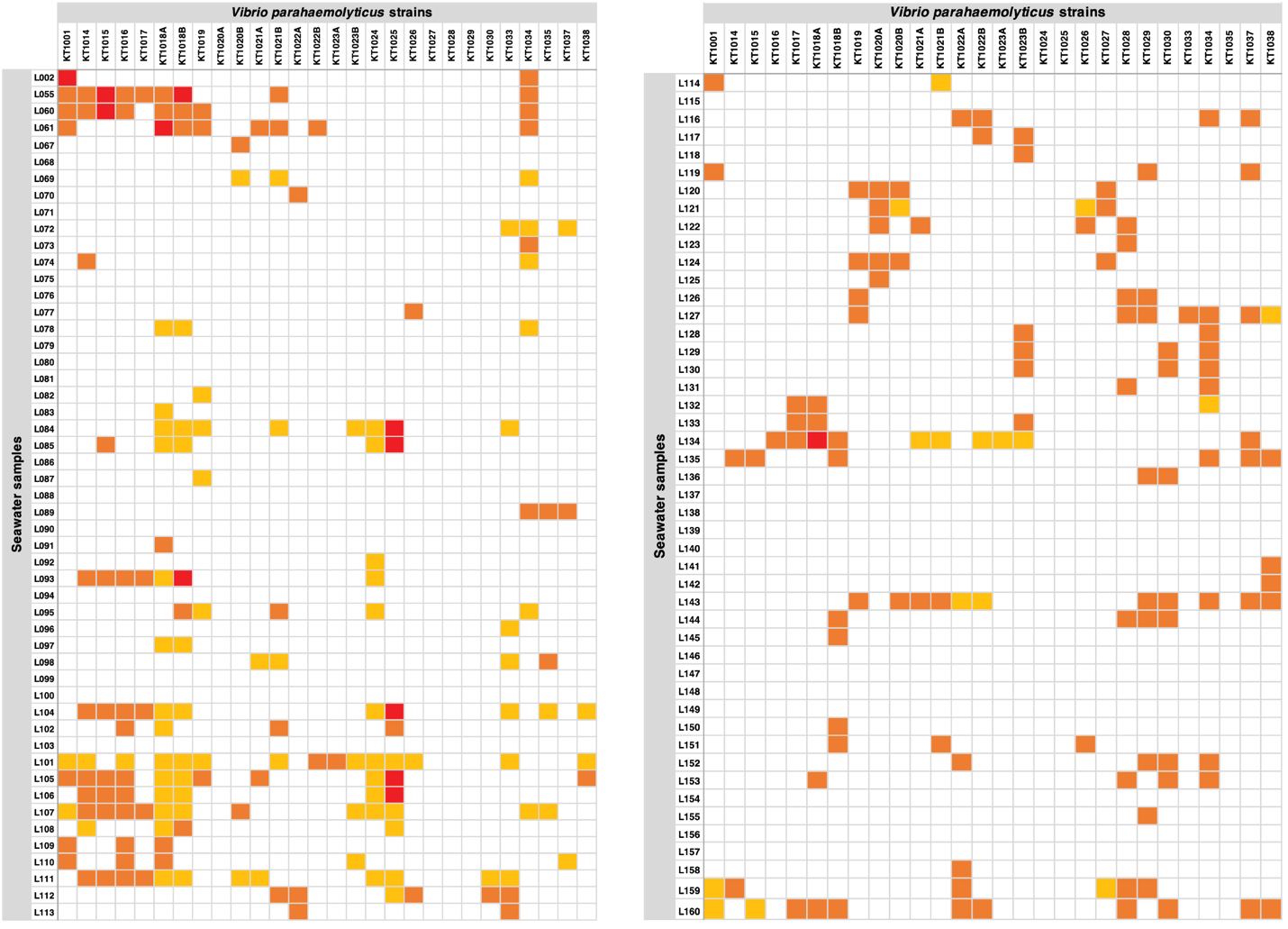
**

**Table S2** Characteristics of *Vibrio parahaemolyticus* strains used in this study.

| **Strain no.** | **Source strain** | **Isolated source** | **PCR result** | | | **AHPND** | **Shrimp mortality (%)** |
| --- | --- | --- | --- | --- | --- | --- | --- |
|  |  |  | **Vp-flaE** | **Tumsat Vp1** | **Tumsat Vp3** |  |  |
| KT1001 | TM | Chanthaburi | +ve | +ve | +ve | ✓ | 100 |
| KT1014 | J36 | Songkhla | +ve | +ve | +ve | ✓ | 100 |
| KT1015 | J37 | Songkhla | +ve | +ve | +ve | ✓ | 87.5 |
| KT1016 | J39 | Songkhla | +ve | +ve | +ve | ✓ | 100 |
| KT1017 | J41 | Songkhla | +ve | +ve | - ve | *X* | 100 |
| KT1018 A/B | J42 | Songkhla | +ve | +ve | - ve | *X* | 77.5 |
| KT1019 | ESKF17/35 | Songkhla | +ve | +ve | +ve | ✓ | 100 |
| KT1020 A/B | VPS2 | Rayong | +ve | +ve | +ve | ✓ | 100 |
| KT1021 A/B | SG091 | Phuket | +ve | +ve | +ve | ✓ | 95 |
| KT1022 A/B | Tawach 7B | Songkhla | +ve | +ve | +ve | ✓ | 85 |
| KT1023 A/B | Rayong 9 | Rayong | +ve | +ve | +ve | ✓ | 95 |
| KT1024 | VPP1 | Pattani | +ve | +ve | +ve | ✓ | 100 |
| KT1025 | ESKF06 | Songkhla | +ve | +ve | +ve | ✓ | 100 |
| KT1026 | J43_11 | Rayong | +ve | +ve | - ve | *X* | 27.5 |
| KT1027 | KB3 | Krabi | +ve | +ve | +ve | ✓ | 42.5 |
| KT1028 | KB4 | Krabi | +ve | +ve | +ve | ✓ | 32.5 |
| KT1029 | KB5 | Krabi | +ve | +ve | +ve | ✓ | 32.5 |
| KT1030 | KB6 | Krabi | +ve | +ve | +ve | ✓ | 40 |
| KT1033 | SVP012 | Songkhla | +ve | +ve | +ve | ✓ | 20 |
| KT1034 | SVP014 | Songkhla | +ve | +ve | +ve | ✓ | 47.5 |
| KT1035 | SVP019 | Songkhla | +ve | +ve | +ve | ✓ | 20 |
| KT1037 | SVP026 | Songkhla | +ve | +ve | +ve | ✓ | 50 |
| KT1038 | SVP027 | Songkhla | +ve | +ve | +ve | ✓ | 47.5 |

**Table S3.** Oligonucleotides used in this study

| **Primer** | **Sequence** |
| --- | --- |
| oKT025 | 5’- TCT AGA GTC GAC CTG CAG GCA TGC -3' |
| oKT026 | 5’- GAA TTC GCT AGC CCA AAA AAA CGG GTA TGG -3' |
| oKT027 | 5’- TTT TTT GGG CTA GCG AAT TCT TTG TTT AAC TTT AAG AAG GAG -3’ |
| oKT028 | 5’- GCC TGC AGG TCG ACT CTA GAT CAT TTA TAC AGT TCA TCC ATG -3’ |
| oKT033 | 5'- GCT GCA GGA GGC AGC CAA AAA ATG ATC TCA AAT CGT AAT GAA TG-3' |
| oKT034 | 5'- GCC TGC AGG TCG ACT CTA GATTAC GCG TAA GTA ATA CCA G -3' |
| oKT035 | 5'- GCT GCA GGA GGC AGC CAA AAA ATG ATC TCA GGC AAA AAC G -3' |
| oKT036 | 5'- GCC TGC AGG TCG ACT CTA GAT TAT GCG TAA GTG ATA CCA G -3' |
| oKT042 | 5'- TTT TTG GCT GCC TCC TGC AGC GGC CGC TCC GGA TTT ATA CAG TTC ATC CAT GCC -3' |

**Table S4.** Plasmids constructed for this study

| **Plasmid** | **Description (DNA insert/Vector)** | **Source** |
| --- | --- | --- |
| pKT1125 | sfGFP / pBAD33 | This study |
| pKT1140 | sfGFP-linker-ChmA ORF203 (phiKT1019)/pBAD33 | This study |
| pKT1144 | sfGFP-linker-ChmA ORF180 (phiKT1028)/pBAD33 | This study |
| pVC1148 | sfGFP / pHERD30T | Nguyen, 2021^75^ |

**Table S5.** Plasmid construction

| **Plasmid** | **Description of construction** |
| --- | --- |
| pKT1125 | The primer oKT1027 and oKT1028 were used to amplify sfGFP insert from pVC1148. While, the backbone of pBAD33 was amplified by using the primer oKT1025 and oKT1026. These 2 amplicons were assembled together by NEBuilder® HiFi DNA Assembly Cloning Kit (New England Biolabs). |
| pKT1140 | The ChmA of vibriophage Eric (phiKT1019) was amplified as an insert from genome of vibriophage Eric by the primers of oKT1033 and oKT1034. The backbone was amplified from pKT1125 by the primer oKT1025 and oKT1042. These 2 amplicons were assembled together by NEBuilder® HiFi DNA Assembly Cloning Kit (New England Biolabs). |
| pKT1144 | The sfGFP-fused ChmA recombinant plasmid was constructed same as above. However, the insert of ChmA of vibriophage Ariel (phiKT1028) was amplified from genome of vibriophage Ariel by the primer oKT1035 and oKT1036. |

**Table S6.** The recombinant *V.parahaemolyticus* strains used in this study

| **Strain number** | **Plasmid name** | **Phage** | **Phage Protein** | **Host** |
| --- | --- | --- | --- | --- |
| KT1135 | 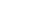pKT1140 | phiKT1019 | sfGFP-ChmA (ORF203 of phiKT1019) | KT1018B |
| KT1139 | pKT1144 | phiKT1028 | sfGFP-ChmA (ORF180 of phiKT1028) | KT1018B |
| KT1145 | pKT1125 | - | sfGFP | KT1018B |
